# Bovine Serum Albumin Detection by Graphene Oxide Coated Long-Period Fiber Grating

**DOI:** 10.1007/s13320-022-0649-6

**Published:** 2022-01-18

**Authors:** Ruiduo Wang, Hao Wu, Mei Qi, Jing Han, Zhaoyu Ren

**Affiliations:** 1 The State Key Laboratory of Photoelectric Technology and Functional Materials, International Collaborative Center on Photoelectric Technology and Nano Functional Materials, Institute of Photonics & Photon Technology, Northwest University, Xi’an, 710127 China; 2 The State Key Laboratory of Transient Optics and Photonics, Xi’an Institute of Optics and Precision Mechanics, Chinese Academy of Sciences, Xi’an, 710119 China; 3 The School of Information Science and Technology, Northwest University, Xi’an, 710127 China

**Keywords:** Graphene oxide, bovine serum albumin, biosensor

## Abstract

A biosensor for bovine serum albumin (BSA) detection by graphene oxide (GO) functionalized micro-taped long-period fiber grating (GMLPG) was demonstrated. The amide bond connected between the GO and BSA enabled the BSA to attach onto the fiber surface, which changed the effective refractive index of the cladding mode and characterized the concentration of the BSA. This real-time monitoring system demonstrated a sensing sensitivity of 1.263 nm/(mg/mL) and a detection limit of 0.043 mg/mL. Moreover, it illustrated superior measurement performance of higher sensitivity in the presence of glucose and urea as the interference, which showed static sensitivities of ∼1.476 nm/(mg/mL) and 1.504 nm/(mg/mL), respectively. The proposed GMLPG demonstrated a great potential for being employed as a sensor for biomedical and biochemical applications.

## 1. Introduction

Serum albumin (SA) is the most widely investigated transparent protein among all kinds of proteins in organisms. It is abundant in the plasma of organisms and contributes nearly 80% of the osmotic pressure [1]. In addition to maintaining osmotic pressure, it also exhibits a variety of biological functions, such as buffering pH, providing nutrients, transporting carriers, scavenging free radicals, and inhibiting platelet aggregation and anticoagulation [2–4]. In healthy individuals, the SA concentration ranges from 35 g/L to 55 g/L, and the aberrant amount and abnormal component content of SA are closely associated with various diseases [5, 6]. Therefore, SA detection is of great importance in clinical practice and recommended for disease diagnosis. The widely accepted detection methods include agarose electrophoresis, cellulose acetate membrane electrophoresis, and capillary electrophoresis-ultraviolet. Among these methods, the minimum detection limit is $10^{-7}$ mol/L – $10^{-8}$ mol/L [7]. Bovine serum albumin (BSA), which has 76.5% homology with human serum albumin, is often used as a model biomolecule for researching related works owing to its low price.

Due to the high sensitivity to the environmental refractive index (RI), etched Bragg fibers gratings and long-period fiber gratings (LPGs) are commonly used in the detector structure [8, 9]. Graphene, as the first two-dimensional material to be exfoliated, has been widely researched in the detector applications owing to its superior optical and optoelectronic properties. Several addressed works of the detector with the optical fiber combined graphene structure are mostly used for sensing in the fields of mechanics [10], electricity [11], chemical temperature [12, 13], gas concentration [8], and biology. Especially in the biological field, the traditional detection methods have encountered some problems, such as a large amount of sampling, post-sampling cultivation, time consuming, or low sensitivity. A variety of efforts have been made to detect biochemical substances in the medical research fields. In the actual medical process, it is necessary to obtain the test results easily and quickly. Through the real-time display of spectra for the fiber detector, combined with graphene oxide (GO) or other surface functional materials, it can improve the overall sensitivity of the device [9]. Due to the biocompatibility and large number of binding sites on the surface for further modification, the detector based on graphene covered on etched nano fiber, the Bragg fiber grating or long-period fiber grating could be used to test heavy metal elements [14, 15], hemoglobin [16, 17], C-reactive protein [18, 19], viruses [20, 21], bacteria [22], BSA [23, 24], glucose [25], etc.

In this paper, we demonstrated a sensitive biosensor for the detection of the BSA by the GO deposited on the micro-taped long-period fiber grating (GMLPG). The GMLPG was characterized by the scanning electron microscope (SEM), atomic force-microscopy (AFM), and Raman spectroscopy to reveal the surface morphology and coated GO sheets. By employing the carboxyl group in the GMLPG, the concentration of the BSA can be monitored in real time. In addition, concerning the complexity of latent samples, the concentration of the BSA was also investigated in the presence of urea and glucose. This work indicated the biosensor based on the GO has high sensitivity and could be used for biochemical detection.

## 2. Experimental method

### 2.1 Chemicals and materials

The experiment employed graphite, concentrated sulfuric acid, concentrated nitric acid, potassium permanganate, hydrogen peroxide, methanol, ethanol, acetone, isopropanol, deionized (DI) water, sodium hydroxide (NaOH), (3-Aminopropyl) triethoxysilane (APTES), BSA, N-hydroxysuccinimide (NHS), 1-(3-Dimethylaminopropyl)-3-ethylcarbodiimide (EDC) hydrochloride, and diluted hydrochloric acid. All chemical reagents used in the experiment were analytically pure and could be used directly without further purification.

### 2.2 Principle and fabrication of the GMLPG

The micro-taped long-period fiber grating (MLPG) was made by the standard single-mode fiber (SMF). During the fabrication process, the SMF was locally heated by a carbon dioxide laser (the laser power was 6 W and the exposure duration was approximately 4 s). Subsequently, the heated region was pulled by two fiber holders that were installed on the stepping motors. To fabricate the MLPG, the pulling device performed the elongation process, and the laser was split into two beams, which could ensure that the two counter parts of the SMF could be heated uniformly. During the fabricating process, a broadband light source (BBS) and an optical spectrum analyzer (OSA) were utilized to monitor the transmission spectrum. Once the 4 s heating process finished, a taper was created by the stepping motors, which stretched the fiber in the same direction with a different speed. An MLPG was typically formed by a periodic RI modulation in the order of hundreds of micrometers in the fiber. The pertubation of RI induced light coupled from the core mode to the forward-propagating cladding modes and induced a series of attenuation bands in the spectrum. The resonant wavelength satisfied the phase-matching condition as follows [26]:

$$\lambda_{\text{res}} = \left(n_{\text{eff}}^{\text{co}} - n_{\text{eff}}^{\text{cl,m}}\right)\Lambda \tag{1}$$

where $\lambda_{\text{res}}$ is the coupling central wavelength; $n_{\text{eff}}^{\text{co}}$ and $n_{\text{eff}}^{\text{cl,m}}$ are the effective RIs of the core mode and m-order cladding mode, respectively; $\Lambda$ is the grating period. Additionally, $T$ denotes the function of the grating length and is given by [26]

$$T = 10\lg\left[\cos^2\left(kL\right)\right] \tag{2}$$

where $L$ ($L = N\Lambda$) is the length of the periodical MLPG, $N$ is the tapered number, and $k$ denotes the coupling coefficient. To control the high-order resonant cladding mode at around 1 550 nm, the transmission spectrum of the MLPG was real-time monitored. The period of the fabricated MLPG was ~680 μm, the waisted diameter was ~109 μm, and $N$ was 40. The high-order mode could be analyzed to be $LP_{14}$ cladding mode according to the charge coupled device (CCD) image of the field distribution, which presented four concentric rings totally [27]. The proposed MLPG exhibited significant sensitivity of –165.5 nm/RIU itself by sensing the RI of the sucrose solution, which gave the further potential for GO functionalization, and it featured with low transmission loss, without annealing, superior compatibility, and low cost.

The GO was prepared by the modified Hummers method [28]. The process that attached the GO onto the fiber is shown in Fig. 1. Firstly, the MLPG was cleaned by the acetone solution to remove the residue on the surface. After that, the MLPG was immersed in the 1.0 M sodium hydroxide solution for 1 hour to make –OH bond adhere to fiber's surface, then it was washed by DI water and dried. Subsequently, the MLPG was immersed in the 5% APTES solution for 1 hour to generate Si–O–Si bonds on the surface, and then the unbonded monomers were washed away by ethanol. Then, the optical fiber was dried at 70 ℃ for 30 minutes to improve the stability of APTES. After that, a BBS that ranged from 1 520 nm to 1 570 nm was injected into the MLPG to monitor the resonant wavelength ($\lambda_{\text{res}}$), and the OSA (Yokogawa AQ 6370) was employed to monitor the transmission spectrum. Compared with the deposition by chemical bonding methods [16], the optical tweezer effect and heating were employed to further improve the efficiency and deposition stability. The grating area was placed in the V-shape groove with a heating plate (maintained at 50 ℃) below to intensify the molecular movement. The deposition process was carried out by filling the GO supernatant in the V-shape groove and coated around the grating region, which simultaneously injected a pump light (980 nm and 120 mW) into the fiber to produce the optical tweezer effect, in order to generate the photodynamic properties and force GO nanosheets towards the evanescent field region. As a result, the 2-hour GO deposition process was done and followed by thoroughly drying to ensure the surface stability.

### 2.3 Surface morphological characterization

To characterize the prepared GO, the GO solution was filtered and this GO film was characterized by the SEM, as shown in Fig. 2(a). The observation area (the size of ~100 μm$^2$) illustrated that the GO was transformed onto its surface in a few layer structures, which demonstrated well surface characteristics and no bulk material structure appeared. In addition, the Raman spectrum of the GO was recorded on a Raman micro-spectrometer with 532 nm laser, as depicted in Fig. 2(b). The Raman spectrum of the GO consisted of two prominent bands, the defect D-band at 1 360 cm$^{-1}$

and G band at 1 570 cm$^{-1}$, which were basically consistent with previous report [29].

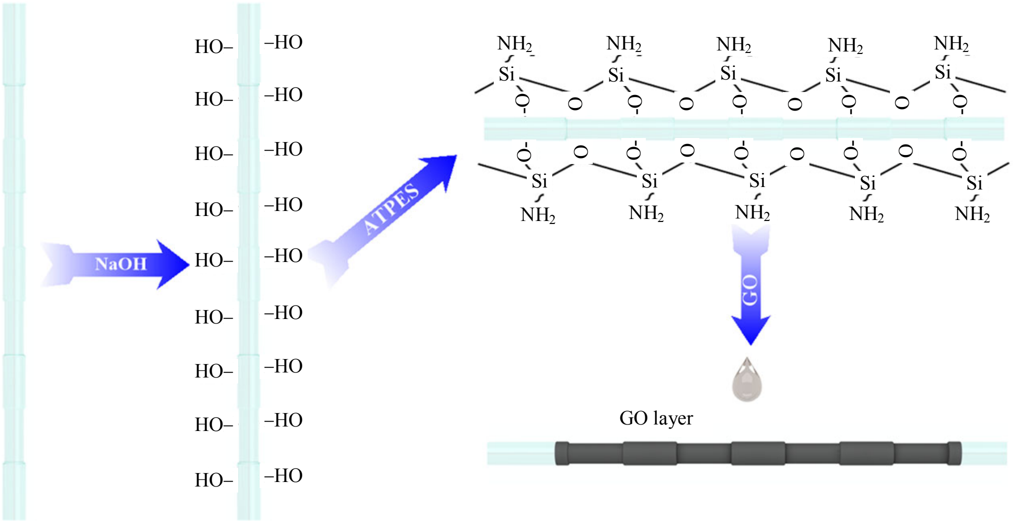

Fig. 1 Schematic diagram of the fabrication process of the GMLPG.

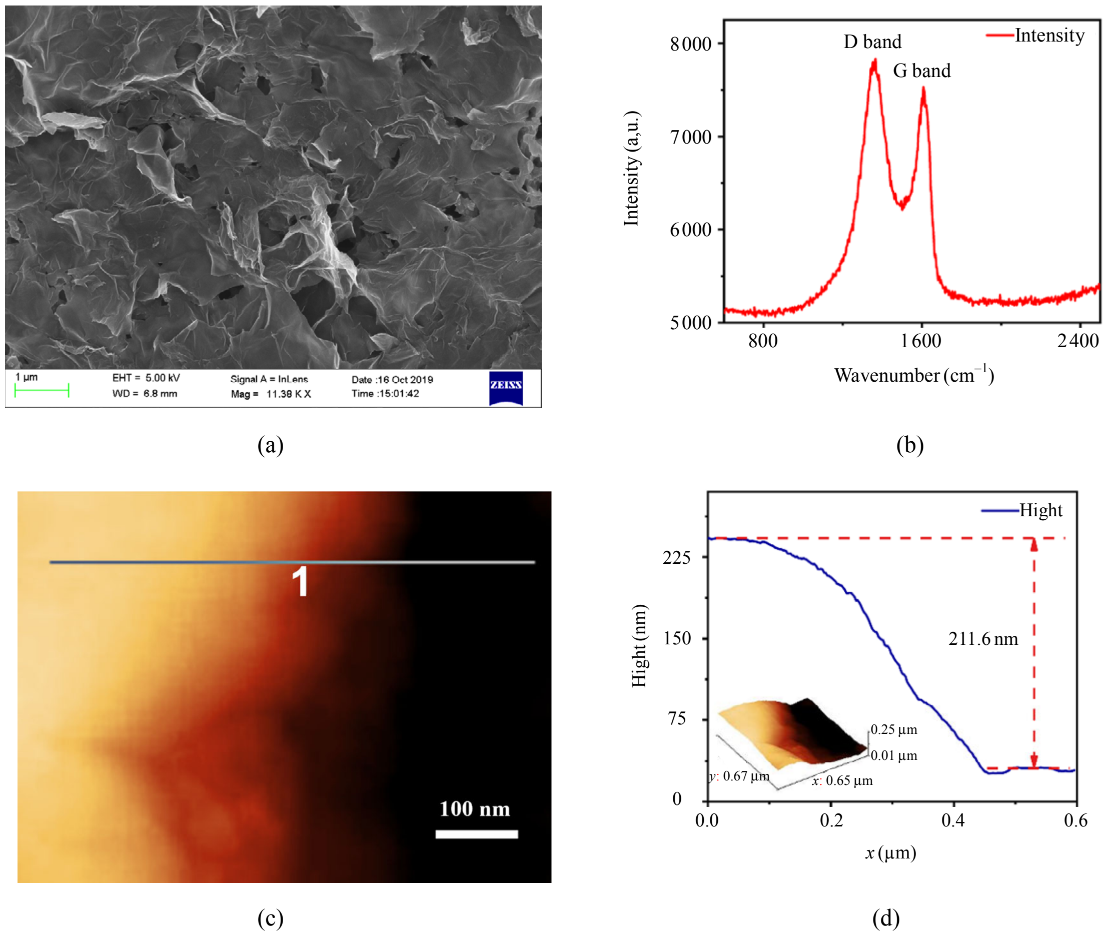

Fig. 2 Characterization of the GO and GMLPG: (a) SEM image of the GO, (b) Raman spectrum of the GO, (c) AFM image of the GMLPG, and (d) height variations at the edge of the deposited GO.

In order to measure the thickness of the GO that was deposited on the MLPG, the AFM measurement was done at the edges of the grating region, the tapping mode AFM togographic image, and height profile as shown in Figs. 2(c) and 2(d), respectively. The results showed that the thickness of the deposited GO was identified to be ~211.6 nm.

## 3. Experimental results and discussion

### 3.1 Interrogation setup

The biosensing interrogation system that features with real-time monitoring is shown in Fig. 3. A continuous BBS was employed to be injected into the GMLPG and the output light was monitored by the OSA. In order to avoid the interference of tension and temperature, the GMLPG was placed onto the V-shape groove and fixed on a pair of fiber holders with no initial torsion or bend. The temperature of environment was maintained within the range of (23±1) ℃ owing to that the MLPG was sensitive to the environment temperature, and a minor temperature variation might cause cross-sensitivity. The temperature sensitivity could be compensated and offset according to results in the previous work [12]. We configured the BSA as aqueous solutions with different gradient concentrations (0.4 mg/mL, 0.8 mg/mL, 1.2 mg/mL, 1.6 mg/mL, and 2.0 mg/mL, respectively) and each solution sample contained 2 mg EDC and 4 mg NHS. Each measurement of BSA samples was dropped into the grating area for 3 minutes and the change of the $\lambda_{res}$ ($LP_{14}$ cladding mode) was observed by the OSA. After each measurement, the device was washed with dilute hydrochloric acid to remove the BSA that was bound to the GO. The chemical-bonding process would happen once the device was dropped into samples as well as continuous spectrum change. In general, this accumulation process was more efficient during the high-concentration sample detection. In order to calibrate this accumulation over time without redundant variable, reaction time of 3 minutes was determined and real-time monitoring could verify the effectiveness of the result during the process.

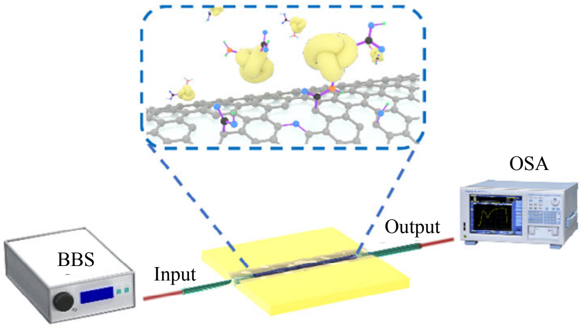

Fig. 3 Measurement system for testing the BSA by using the GMLPG.

### 3.2 BSA detection

The spectra of the MLPG and GMLPG of BSA measurement with different concentrations are shown in Figs. 4(a) and 4(b), respectively. As the BSA concentration increased, $\lambda_{res}$ of the $LP_{14}$ mode of the two groups of devices appeared in different orders of blue shift. Under the same concentration difference, the $\lambda_{res}$ variation ($\Delta\lambda_{res}$) of the GMLPG group was significantly higher than that of the MLPG group. The data analysis by linear fitting illustrated that as the BSA concentration increased both $\lambda_{res}$ variations of the MLPG and GMLPG were enhanced, as shown in Figs. 4(c) and 4(d), respectively. In the linear fitting result with excellent $R^2$ coefficient of approaching 1, the slope value $K$ was the static sensitivity of the device [30], where the values of the MLPG and GMLPG were 0.221 nm/(mg/mL) and 1.263 nm/(mg/mL), respectively. Obviously, by GO deposition, the sensitivity of the device was increased by ~5.7 times. $\delta_L$ is the linearity of the device, which is defined as follows [30]:

$$\delta_L = \pm\frac{\Delta Y_{\max}}{Y_{\text{F-S}}}\times 100\% . \tag{3}$$

where $\delta_L$ is calculated to be 4.1%, $\Delta Y_{\max}$ is the the maximum deviation between the experimental data and the fitting curve, and $Y_{\text{F-S}}$ is the full-scale output of the device. Owing to that the minimum resolution of the OSA measurement system was 0.02 nm and the resolution for BSA detection was

0.043 mg/mL from the linear fitting curve, the results are considered to be valid.

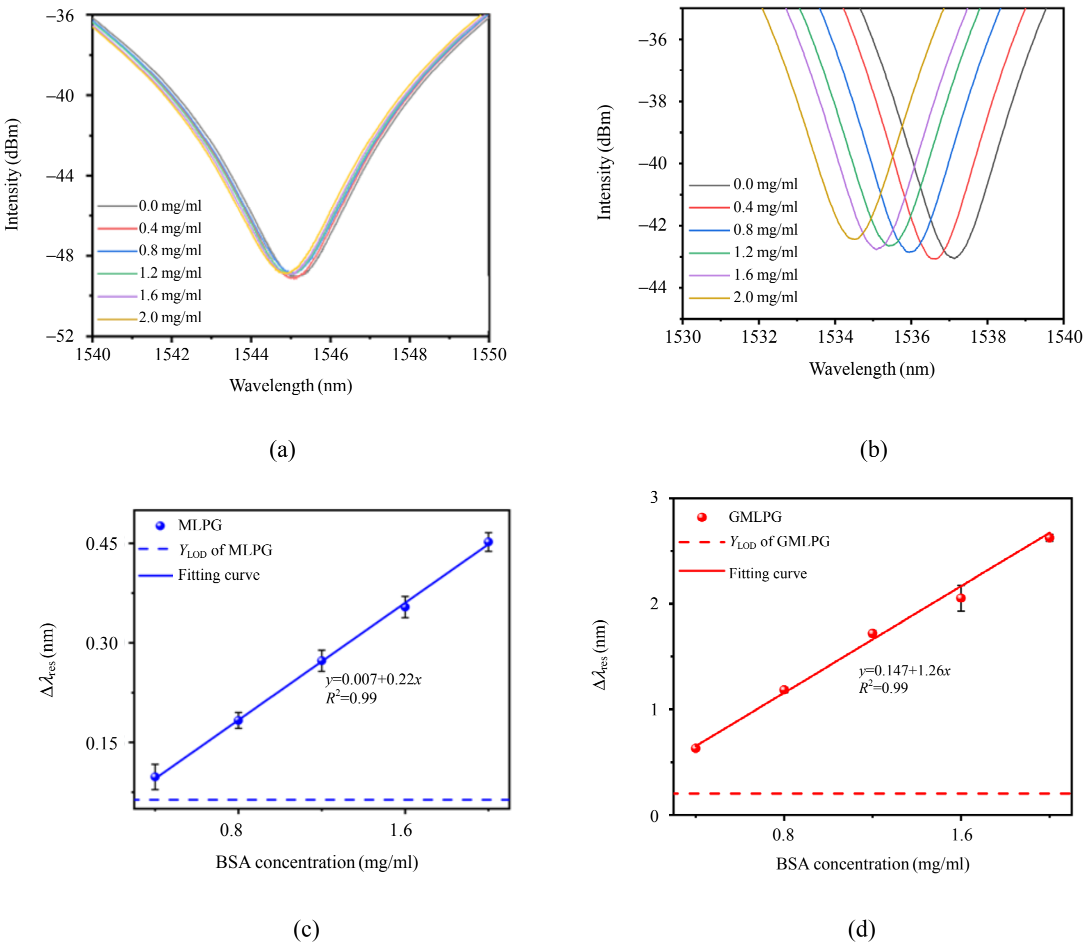

Fig. 4 Sensing spectrum and fitting results: (a) and (b) are the transmission spectra of the MLPG and GMLPG with different BSA concentrations; (c) and (d) are the dependences of $\Delta\lambda_{res}$ against the BSA concentration in DI water by the MLPG and GMLPG, respectively. The dash horizontal lines represent the three times of the standard deviation of blank measurement.

The limit of detection (LOD) of the measurements was determined by investigating the standard deviation of the low-concentration sample [31]. $Y_{LOD}$ could be calculated by adding the average value of $y$ at the blank sample concentration with multiple measurements and three times of the standard deviation measured at the low concentration [31]. $Y_{LOD}$ of the MLPG and GMLPG was calculated to be 0.063 nm and 0.202 nm, respectively. Similarly, conversed by the relationship, $X_{LOD}$ of the MLPG and GMLPG was calculated to be 0.256 mg/mL and 0.043 mg/mL, respectively. It could be seen that with GO coated, the GMLPG could effectively reduce $X_{LOD}$ of the sensor, which was ~6 times lower than that of the MLPG. In addition, the standard relative error of the MLPG measurement in the same concentration of the 0.4 mg/ml group was 19.24%, which was significantly higher than that in the GMLPG group (2.88%). Obviously, the MLPG group had the low reliability at low concentrations. In the relative uncertainty of the un-deposited GO group, compared with uncertainty of the OSA, the data uncertainty was the main factor, which ensured the reliability of GMLPG group results. In other words, the uncertainty as well as $X_{LOD}$ would further decrease when the higher-precision OSA was employed.

The coupling central wavelength of the LPFG was determined by RIs of the core propagation mode and the cladding mode [32]. The effective refractive index of the *m*-order cladding mode was determined by RI of the cladding as well as the surrounding medium. The carboxyl functional groups on the GO surface would automatically combine with amino in the BSA, which enabled the BSA to be attached on the GO surface by the connection of the amide bond, as shown in Fig. 3. The NHS and EDC added in the solution could enhance the formation of amide bonds, which enabled the BSA to better adhere onto the surface of fiber and changed the effective RI of the cladding mode of the LPFG. Furthermore, $\lambda_{\text{res}}$ drifted with the change of BSA concentrations. With reference to the core RI of the SMF, it could be calculated when the BSA solution of 0.4 mg/mL was added, the effective RI of the cladding mode increased $8.2\text{e}^{-7}$ due to the amide bond attaching BSA to the GO surface. After each measurement, the hydrochloric acid cleaning could effectively break the amide bond and remove the residue.

### 3.3 Sensing in the presence of interfering compounds and reusability

Considering the complex compounds in the biological system, furthermore, the response of the BSA analyte in the presence of urea or glucose as interfering compounds has been carried out. The concentrations of urea and glucose were 1 800 mg/L and 3 800 mg/L respectively, which were higher than the normal ranges in the human body [33]. By repeating the previous experiment of measuring the BSA sample with the concentration of 0.0 mg/mL – 2.0 mg/mL, it was observed that both in urea and glucose solution, $\Delta\lambda_{\text{res}}$ demonstrated the same blueshift tendency as the BSA concentration increased as shown in Figs. 5(a) and 5(b), respectively. According to $\Delta\lambda_{\text{res}}$ as shown in Fig. 5(c), the linear fitting results illustrated the sensitivities of 1.476 nm/(mg/mL) and 1.504 nm/(mg/mL) in the presence of glucose and urea, respectively. The corresponding LODs of the GMLPG were 0.029 mg/mL and 0.032 mg/mL in the presence of urea and glucose, respectively, which were lower than the results without interfering compounds.

Comparing these results with the non-interference group in the previous section, the results of the GMLPG in the presence of glucose and urea could also improve the sensitivity in the same concentrations. $n_{\text{eff}}^{\text{cl,m}}$ in (1) was determined by the difference between RIs of the cladding and surrounding medium (SRI). $\Delta\lambda_{\text{res}}$ with respect to SRI is [19]

$$\frac{\mathrm{d}\lambda_{\text{res}}}{\mathrm{d}n_{\text{sur}}} = \frac{\mathrm{d}\lambda_{\text{res}}}{\mathrm{d}n_{\text{eff}}^{\text{cl,m}}} \cdot \frac{\mathrm{d}n_{\text{eff}}^{\text{cl,m}}}{\mathrm{d}n_{\text{sur}}} = \lambda_{\text{res}} \cdot \gamma \cdot \varGamma_{\text{RI}} \tag{4}$$

where $n_{\text{sur}}$ is the RI of the surrounding medium, $\gamma$ is the waveguide dispersion, and $\varGamma_{\text{RI}}$ is the dependence of the waveguide dispersion in the environment, which is given by [34]

$$\varGamma_{\text{RI}} = -\frac{u_m^2 \lambda_{\text{res}}^3 n_{\text{sur}}}{8\pi^3 r_{\text{cl}}^3 n_{\text{cl}} \left(n_{\text{eff}}^{\text{co}} - n_{\text{eff}}^{\text{cl,m}}\right)\left(n_{\text{cl}}^2 - n_{\text{sur}}^2\right)^{3/2}} \tag{5}$$

where $u_m$ is the *m*-th root of the zero-order Bessel equation $[J(0)_u = 0]$, and $r_{\text{cl}}$ and $n_{\text{cl}}$ represent the radius and RI of the fiber cladding, respectively. The sensing sensitivity was higher when the RI of the SRI was closer to $n_{\text{cl}}$. Under the same condition of the BSA concentration, the addition of glucose or urea made the environmental RI higher than those of the non-interference groups, which was more close to the RI of the fiber core. Therefore, $\Delta\lambda_{\text{res}}$ would further increase at high concentration samples. The experimental results demonstrated that the measurement of the BSA concentration could still be obtained from the drift of the spectral line under interference factors. Recently, the detection of the nonlabelled BSA using photothermal optical phase shift (POPS) detection with ultraviolet (UV) excitation as well as BSA detection by atmospheric pressure plasma jets in matrix-assisted laser desorption ionization time-of-flight mass spectrometry (MALDI-TOF-MS) has been addressed [35, 36]. Therefore, the GMLPG demonstrated a great potential for biosensing due to the simple monitoring system, label-free, and comparable LOD.

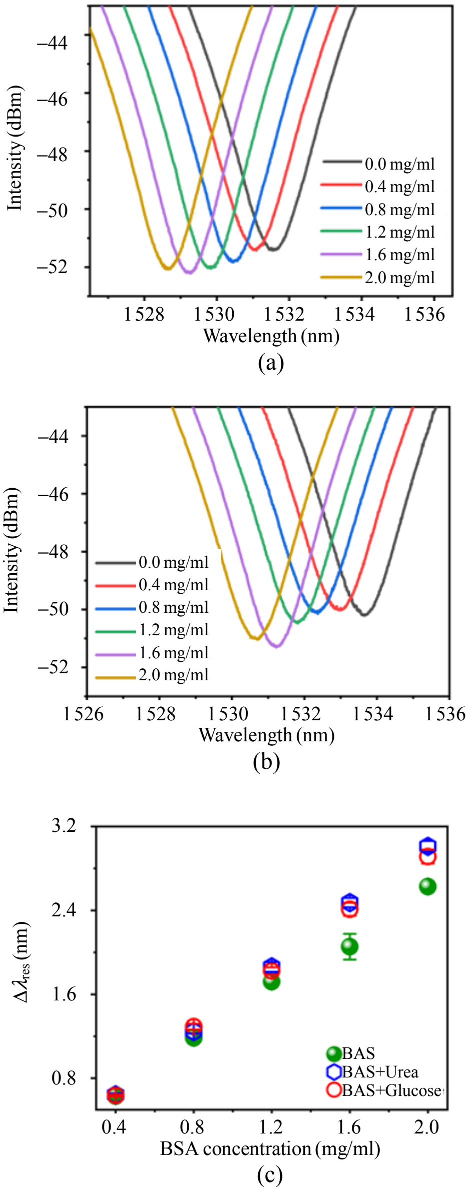

Fig. 5 Sensing results of the GMLPG in the presence of urea and glucose: (a) and (b) are the transmission spectra of the GMLPG with different BSA concentrations in the presence of urea and glucose, respectively; (c) $\Delta\lambda_{res}$ of the GMLPG against the BSA concentration in DI water, urea, and glucose, respectively.

In real applications, the reusability is a crucial function for the biosensor. Therefore, on this basis, the aforementioned results were set as the 1st cycle, and the sensing characteristics of reusability was validated by repeating biosensing measurement of the 2 nd cycle and the 3 rd cycle. We repeated the entire experiment cycles for three times and recorded the total $\Delta\lambda_{res}$ with concentration changes from 0.0 mg/mL to 2.0 mg/mL in the presence of urea, glucose, and only DI water, respectively. After each cycle, the GMLPG and V-shape groove were rinsed with methanol to break the formed bonding and washed for ten minutes and dried. The compared results of three cycles when measuring the 0.4 mg/mL and 2.0 mg/mL BSA samples are presented in Figs. 6(a) and 6(b), respectively. The results simply displayed minor fluctuations in various cycles and demonstrated the similar wavelength drift at the same concentration on the whole. The lower $\Delta\lambda_{res}$ in the 2 nd and 3 rd cycles illustrated the minor residue and the GO layer was not absolute stable. Whereas, the whole sensing tendency and error were within the acceptable range, and no distinct variation was obtained when monitoring samples with different interfering compounds.

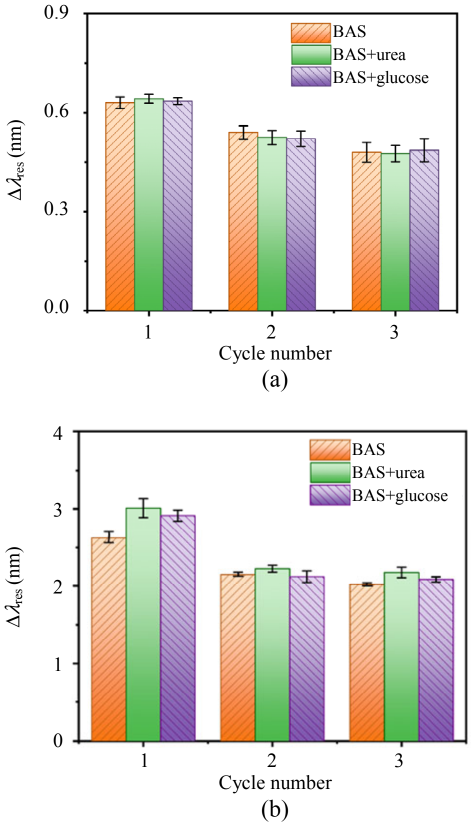

Fig. 6 Repeatabilities of the wavelength shift when measuring (a) the BSA analyte (0.4 mg/mL) and (b) BSA (2.0 mg/mL) during the three cycles, respectively.

## 4. Conclusions

GO nanosheets have been coated uniformly with the required thickness on the MLPG surface using the chemical bonding and optical tweezer technology. The SEM, AFM, and Raman spectra have characterized the surface morphology of the GO layer and illustrated a desirable thickness of 211.6 nm. A case study of the BSA detection by the GMLPG has displayed a phenomenal increase in the sensitivity and LOD compared to the bare MLPG. In addition, it still exhibited excellent measurement performance and higher sensitivity in the presence of glucose and urea as interfering components. The static sensitivity was 1.263 nm/(mg/mL) and the LOD of the GMLPG in DI water, urea, and glucose were 0.043 mg/mL, 0.029 mg/mL, and 0.032 mg/mL, respectively. The sensor has shown quick detection time, good reusability, and stability. Owing to the inherently excellent optical and biochemical properties, the GO nanosheets as a bio-interface material could provide the enhanced sensitivity and high efficiency. MLPG's inherent qualities, such as low transmission loss and multiplexing capability, make the GO-MLPG an attractive contender as a label-free and cost-effective device for large-scale production, which has a potential for biochemical applications.

## Acknowledgment

This work was supported by the International Cooperative Program (Grant No. 2014DFR10780), the National Science Foundation of China (Grant No. 11874299), and the Science and Technology Innovation and Entrepreneurship Double Tutor Project of Shaanxi Province (Grant No. 2018JM1059). The authors would like to thank Professor Zhengquan HE for his support in experimental conditions.

**Open Access** This article is distributed under the terms of the Creative Commons Attribution 4.0 International License (http://creativecommons.org/licenses/by/4.0/), which permits unrestricted use, distribution, and reproduction in any medium, provided you give appropriate credit to the original author(s) and the source, provide a link to the Creative Commons license, and indicate if changes were made.
